# Lupin Seed Protein Extract Can Efficiently Enrich the Physical Properties of Cookies Prepared with Alternative Flours

**DOI:** 10.3390/foods9081064

**Published:** 2020-08-05

**Authors:** Joana Mota, Ana Lima, Ricardo B. Ferreira, Anabela Raymundo

**Affiliations:** LEAF—Linking Landscape, Environment, Agriculture and Food, Instituto Superior de Agronomia, Universidade de Lisboa, Tapada da Ajuda, 1349-017 Lisbon, Portugal; agusmaolima@gmail.com (A.L.); rbferreira@isa.ulisboa.pt (R.B.F.); anabraymundo@isa.ulisboa.pt (A.R.)

**Keywords:** white lupin seeds extract, snack, protein, texture, viscoelastic behavior

## Abstract

Legume proteins can be successfully used in bakery foods, like cookies, to obtain a protein-enriched product. A lupin extract (10 g/100 g) was added to gluten and gluten-free flours from different sources: rice, buckwheat, oat, kamut and spelt. The impact on the physical properties of the dough and cookies was evaluated for the different systems. Rice and buckwheat doughs were 20% firmer and 40% less cohesive than the others. The incorporation of lupin extract had a reduced impact on the shape parameters of the cookies, namely in terms of area and thickness. The texture differed over time and after eight weeks, the oat and buckwheat cookies enriched with lupin extract were significantly firmer than the cookies without lupin. The incorporation of lupin extract induced a certain golden-brown coloring on the cookies, making them more appealing: lightness (L*) values decreased, generally, for the cookies with lupin extract when compared to the controls. The a_w_ and moisture content values were very low for all samples, suggesting a high stability food product. Hence, the addition of lupin extract brought some technological changes in the dough and cookies in all the flours tested but improved the final product quality which aligns with the trends in the food industry.

## 1. Introduction

Current trends in the development of new food products identified by companies in consumer studies, such as Innova Market Insights, are gluten-free products, alternative vegetable proteins and snacks. In this context, the snack market is very prominent, with the demand for healthy snacks becoming increasingly relevant [1]. Cookies and crackers have become one of the most popularly consumed snacks due to their low manufacturing cost, availability, high nutrient density, long shelf-life and potential to be supplemented with a wide variety of nutraceuticals [2,3]. It is widely known that wheat cookies, commonly available in the market, lack good quality protein because of their deficiency in lysine. For this reason, the production of wheat cookies with various legume seeds has been proposed [4], to increase the protein content and improve an amino acid balance of the final product, due to the contribution of lysine by legumes and the contribution of methionine by cereals [5]. However, wheat gluten consisting of glutenins and gliadins cause severe intestinal inflammation in individuals suffering with celiac disease or other forms of gluten intolerance [6]. Hence, several alternative flours have an increase in demand, such as spelt and kamut, as is the case for species of the *Triticum* genus, but with a healthier nutritional profile than modern wheats, as they provide more nutraceutical compounds, vitamins and minerals [7,8].

A study performed under in vitro inflamed conditions reported that ancient *Triticum* grains (spelt and kamut) had a less inflammatory activity by decreasing IL-8 production, when compared to the modern grains [8]. Another study showed a better response to oxidative stress in rats fed with kamut bread than with wheat bread [9]. The available data suggest that ancient grains cause less inflammation than modern wheat grains, being an important and healthier alternative as food ingredients for the bakery industry.

Another alternative is oat (*Avena sativa*). The nutritional and health benefits presented by this grain are correlated with an increased intake of β-glucans, polyunsaturated fatty acids, and essential amino acids [10]. β-Glucans in wheat comprise ca. 1% of the seed, but 3 to 7% in oat seeds [11]. The potential use of β-glucans as a food ingredient in functional dietary fiber is increasing. Despite a relatively large quantity of globulins, the proportion of prolamins (like avenin) in oat is lower when compared to the wheat gliadins [12]. In addition, avenins are more easily digested than gliadins. Apparently, the lack of toxic epitopes in avenins compared to gliadins reduce their immunogenicity for celiac patients when compared to wheat prolamins [12]. Nevertheless, oat consumption is only recommended for celiac patients in remission since the contamination of oat by wheat, barley or rye is currently the main limitation for its use in a gluten-free diet [10]. “β-Glucans contribute to the maintenance of normal blood cholesterol levels” (Commission Regulation (EU) 432/2012 of 16 May 2012), is a health claim which highlights the improving health benefits of an oat-containing diet.

The gluten-free flours mostly used by the food industry are rice and buckwheat. Rice flour is a cheap product with a relatively low nutritional value when compared to other gluten-free flours, namely buckwheat, quinoa and maize [13]. The use of buckwheat flour has increased considerably due to the recognition by the consumers of its potential health benefits, presenting an increased commercial interest in the gluten-free market [14]. Nevertheless, several studies showed that many gluten-free foods are deficient in dietary fiber, micronutrients and protein [15,16]. Therefore, the combination of gluten-free flours with other health-promoting factors, such as proteins and bioactive peptides, has also received increased attention as potential functional foods [17].

Legume seeds are food ingredients with high nutritional quality and a low glycemic index when compared to cereal grains [18]. Moreover, legume proteins in the form of flour or concentrate constitute a good supplement for cereal-based foods, because legume and cereal proteins are complementary in their essential amino acids compositions [4,19]. In particular, white lupin (*Lupinus albus)* seeds have received attention as a source of bioactive proteins [20] and have been used as an additive to food products, in order to improve their functional and nutritional properties [19,21]. In 100 g, white lupin contains approximately 32 g protein, 16 g crude fiber, 6 g carbohydrates and 6 g crude fat [22]. Protein content in lupin is higher than in other legumes such as haricot bean, lentil and chickpea, which contain 28.8%, 26.7% and 24.8% protein, respectively [23,24]. For these properties, some researchers used *L. albus* to develop bakery products, such as bread [25], cookies [26] and pasta [21], and the ingestion of lupin-containing foods has been associated with the prevention of diabetes by the hypoglycemic effect, cardiovascular disease, and more recently, digestive tract diseases [20,21,27].

In the available literature, the major studies on lupin-containing foods have been made with flours prepared from the whole seed, therefore containing fat, oligosaccharides, protein and fiber. In addition, there are few or no studies comparing the incorporation of lupin extract (LE) in wheat alternatives such as spelt, kamut, oat and gluten-free (rice and buckwheat) flours. In this sense, the main goal of this study was to evaluate the impact of the addition of LE in the physical properties of sweet cookies prepared with flours (containing or not gluten) of different origins.

## 2. Materials and Methods

### 2.1. Preparation of Lupin Seed Protein Extract

*Lupinus albus* L. sweet seeds were purchased from Jouffray Drillaud, France. Approximately 100 g ± 0.1 g of dry lupin seeds were milled to a powder and extracted using milli-Q water (1:10, *w*/*v*). The extract was stirred overnight at 4 °C. The homogenate was filtered through a miracloth. The final sample was stored frozen a −80 °C overnight and lyophilized (Edwards, Crawley, UK). The final amount of lupin extract (LE) was 50 g [20] and its proximate composition was: 64.07% carbohydrates, 18.06% protein, 7.76% ash, 9.80% moisture and 0.32% lipids. This characterization was performed according to Batista et al. [28], except for the protein content which was quantified by the Bradford method [29].

### 2.2. Flour Composition

Rice flour (Ceifeira, lot L 3411/18, Lisbon, Portugal), oat flour (Próvida, lot 20190910, Lisbon, Portugal), spelt flour (Próvida, lot 20191112, Lisbon, Portugal), kamut khorasan flour (Próvida, lot 20190725, Lisbon, Portugal), buckwheat flour (Próvida, lot 20210630, Lisbon, Portugal) and other ingredients were purchased from a local market. The nutritional composition of the five flours used is summarized in Table 1.

### 2.3. Cookies Preparation

Cookies were prepared according to an optimized formulation [28,30], using the following ingredients (as g/100 g): flour (54), sugar (15), margarine (18), water (12) and baking powder (1). For all the samples, the same quantities of the ingredients were used, except for the flour, which was replaced by 10% (*w*/*w*) LE in the case of lupin cookies. The procedures were similar for the different flours used and the sample without LE incorporation was considered as a control sample for each corresponding flour. The amount of LE to be incorporated in cookies was based on the previous studies [31,32].

Batches of 100 g were prepared, and the ingredients were mixed for 15 s at a speed of 4 in a food processor (Bimby, Vorwerk, Wuppertal, Germany). The sweet cookies were molded in a square mold and baked at 110 °C for 40 min in a forced-air convection oven (Unox, Italy). After cooling for 30 min at room temperature, the cookies were stored in hermetic containers, at room temperature and protected from the light.

### 2.4. Dough Rheology

Rheological measurements were conducted using a controlled strain rheometer (Haake, Mars III, Thermo Fisher Scientific, Karlsruhe, Germany) at a constant temperature (25.0 °C ± 0.1 °C), controlled by a Peltier system. The rheometer was equipped with serrated parallel-plate geometry (20 mm diameter) to overcome the slip effect. The dough pieces were compressed with a 1.5 mm gap. Following the preparation, the dough was allowed to rest for 5 min before measuring. The stress and frequency sweeps were carried out at 25 °C. The stress sweep, with a constant frequency (1 Hz), was performed to identify the linear viscoelastic region. Frequency sweep tests were performed with a constant stress within the linear viscoelastic region and in a frequency range from 0.01 to 100 Hz to obtain the values of elastic modulus (G’ (Pa)) and viscous modulus (G” (Pa)).

### 2.5. Dimensions

The dimensions of the cookies were evaluated using a digital caliper (Powerfix, Germany). The width and thickness of the ten cookies from each formulation were measured after 24 h of cookie preparation.

### 2.6. Color Analysis

The color of the cookie samples was measured using a Minolta CR-400 (Japan) colorimeter. The results were expressed in terms of L*, lightness (values increasing from 0 to 100); a*, redness to greenness (60 to −60 positive to negative values, respectively); and b*, yellowness to blueness (60 to −60 positive to negative values, respectively) according to the CIELab system. The total color difference between the sample cookies during the storage time (up to eight weeks) was determined using average L*, a* and b* values. The measurements were performed under similar light conditions using a white standard (L* = 94.61, a* = −0.53, and b* = 3.62), at room temperature, replicated eight times for each cookie sample (control and lupin-enriched cookies) and for week 0 (24 h after baking) and week 8.

The total colour difference between the control and the lupin-enriched cookies was obtained by Equation (1):

ΔE* = (ΔL* ^2^ + Δa* ^2^ + Δb* ^2^)^1/2^ (1)

### 2.7. Texture Analysis

Instrumental texture analysis was conducted in a TA.XTplus (StableMicro Systems, Godalming, UK) texturometer. Texture measurements were performed at 20 °C  ±  1 °C in a temperature-controlled room.

#### 2.7.1. Dough Texture

Dough samples were submitted to texture profile analyses (TPAs), simulating the action of a double chewing. The dough was contained in a cylindrical flask of 2.5 cm in diameter and 4.5 cm in height. The TPAs were performed in a penetration mode using an acrilic cylindrical probe of 4 mm in diameter, 15 mm of penetration and 1 mm/s of crosshead speed. Firmness and cohesiveness were the two primary texture properties used to compare the doughs, as they were the ones with the greatest capacity to discriminate between the different samples. The firmness of the dough was considered to be the maximum force in the first cycle [33]. The cohesiveness describes how well a food retains its form between the first and second chew and it is a ratio between the work performed in the second and the first cycle [33]. These analyses were repeated eight times for each dough sample.

#### 2.7.2. Cookie Texture

Cookie texture was evaluated with a penetration test, using a cylindrical probe of 2 mm in diameter, plunged 8 mm at 1 mm/s. Resistance to penetration was evaluated by the maximum peak shown on the texturogram which corresponds to the N value. These determinations were replicated at least eight times for each cookie sample (control and lupin-enriched dough) at week 0 (24 h after baking) and week 8.

### 2.8. Water Activity Determination

Cookie samples were analyzed for water activity (a_w_). This was determined using a thermohygrometer (HygroPalm HP23-AW, Rotronic AG, Bassersdorf, Switzerland) at 20 °C ± 3 °C. The tests were performed during storage (24 h and 8 weeks after baking) by crushing the samples into little pieces. The cookies (control and with lupin) were assayed in triplicate.

### 2.9. Moisture Content

The moisture was determined gravimetrically following ISTISAN protocols (ISTISAN Report 1996/34, method B, page 7), using an incubator (Binder GmbH, Germany) at 105 °C until a constant weight was achieved.

### 2.10. Statistical Analyses

Experimental data were obtained at least in triplicate and were statistically analyzed using SigmaPlot (version 12.5). An analysis of variance (one-way ANOVA) was applied to evaluate the differences between samples at a significance level of 95% (*p* < 0.05). Tukey’s test was used to compare the differences between groups. All the results are presented as the mean ± standard deviation (SD).

## 3. Results and Discussion

### 3.1. Physical Properties of the Dough

**Figure 1:**
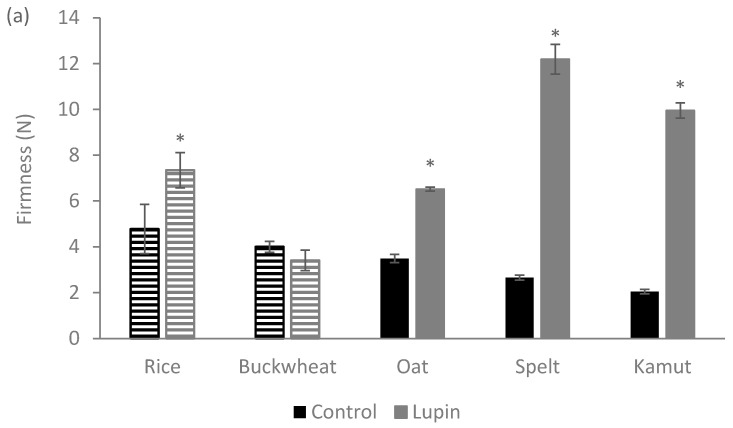

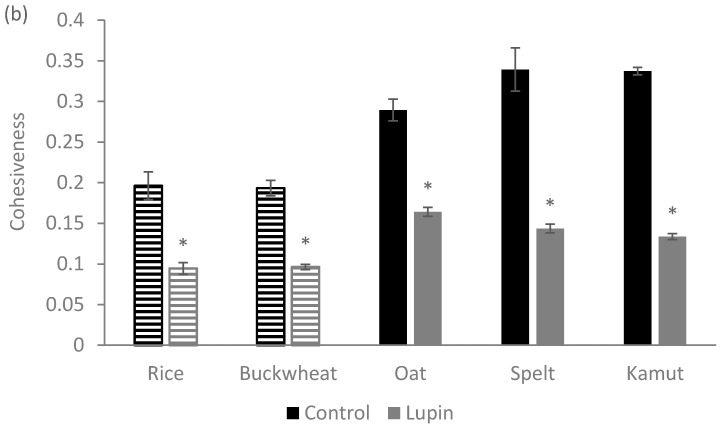
Texture parameters of control and lupin-enriched cookies prepared from five different flours. Solid bars represent the gluten-containing flours (oat, spelt and kamut) and the striped bars represent the gluten-free flours (rice and buckwheat): (**a**) the firmness; and (**b**) the cohesiveness. * represents *p* < 0.05 when compared with the corresponding control cookie.

Figure 1 shows the firmness (a) and cohesiveness (b) of the control dough produced with five flours of different origins and with the corresponding doughs enriched with 10% (*w*/*w*) LE. The doughs prepared with different types of flour have different texture properties (firmness and cohesiveness).

It should be noted that the control gluten-free dough without LE (rice and buckwheat), are 20% firmer and 40% less cohesive than the others. This behavior should result from the different composition of these two flours (Table 1). In these cases, the structuring of the system is essentially achieved by the starch present, although the different types of proteins present can also contribute to the reinforcement of this structure. Thus, the doughs obtained from these two flours have a greater resistance to penetration (high firmness), which is related to more compact doughs. The absence of the gluten matrix decreases the air retention capacity of the system [34], contributing to firmer doughs. At the same time, a reduction in the cohesiveness associated with a greater disaggregation is observed [35]. These characteristics are less positive in terms of the technological handling of these doughs.

In the case of rice, the high starch content is relevant, compared to other flours, which has an important impact on structure creation. Regarding the buckwheat, the type of proteins involved could also explain the increase in firmness and decrease in cohesiveness since its proteins are rich in lysine and arginine, unlike the other flours studied [36]. Complementary studies can be developed, in the future, in order to support this statement.

When 10% (*w*/*w*) of the flours under study is replaced by LE, a relevant impact on the texture characteristics of the dough is observed. In general, the incorporation of proteins contributes to an increase in dough firmness (Figure 1a) and a significant (*p* < 0.05) reduction of at least 50% in cohesiveness (Figure 1b). A similar behavior was observed by the other researchers upon the addition of potato peel to cakes [37], whey protein to cheese [38] and lupin flour to biscuits [26].

It is important to highlight the strong impact of LE addition on the two gluten rich flours—spelt and kamut doughs are about four times firmer (from 2.66 N to 12.19 N, in the case of spelt and from 2.05 N to 9.95 N in the case of kamut) than the corresponding control. This should result from a strong interaction between the main macromolecules present in the system: (i) lupin proteins–flour starch; and (ii) lupin proteins–flour gluten proteins. This type of interactions is strongly dependent on the protein composition of the added protein fraction, as well as on the starch conformation [39]. More important than the total amount of macromolecules present in the dough, which is similar in all the cases, is the biochemical composition and conformation of these proteins and polysaccharides. A firmer dough should reflect a more effective entangled network developed among these macromolecules [39], which may be important in terms of the dough stability, but which translates to a less cohesive dough. 

The relevant reinforcement on the structure observed for the kamut and spelt doughs, due to the incorporation of LE, allows us to predict that there was a reinforcement in the gluten structure already present in the control doughs, resulting from a synergy between the gluten and the lupin proteins. The firmness increase and cohesiveness decrease resulting from the LE incorporation has a relevant impact in technological terms: the doughs become more difficult to mold, meaning it may be necessary to optimize the cookie production process such as the optimization of the water absorption (e.g., MicrodoughLab procedure) that consists of the quantity of water needed to reach the optimal dough consistency [40].

**Figure 2:**
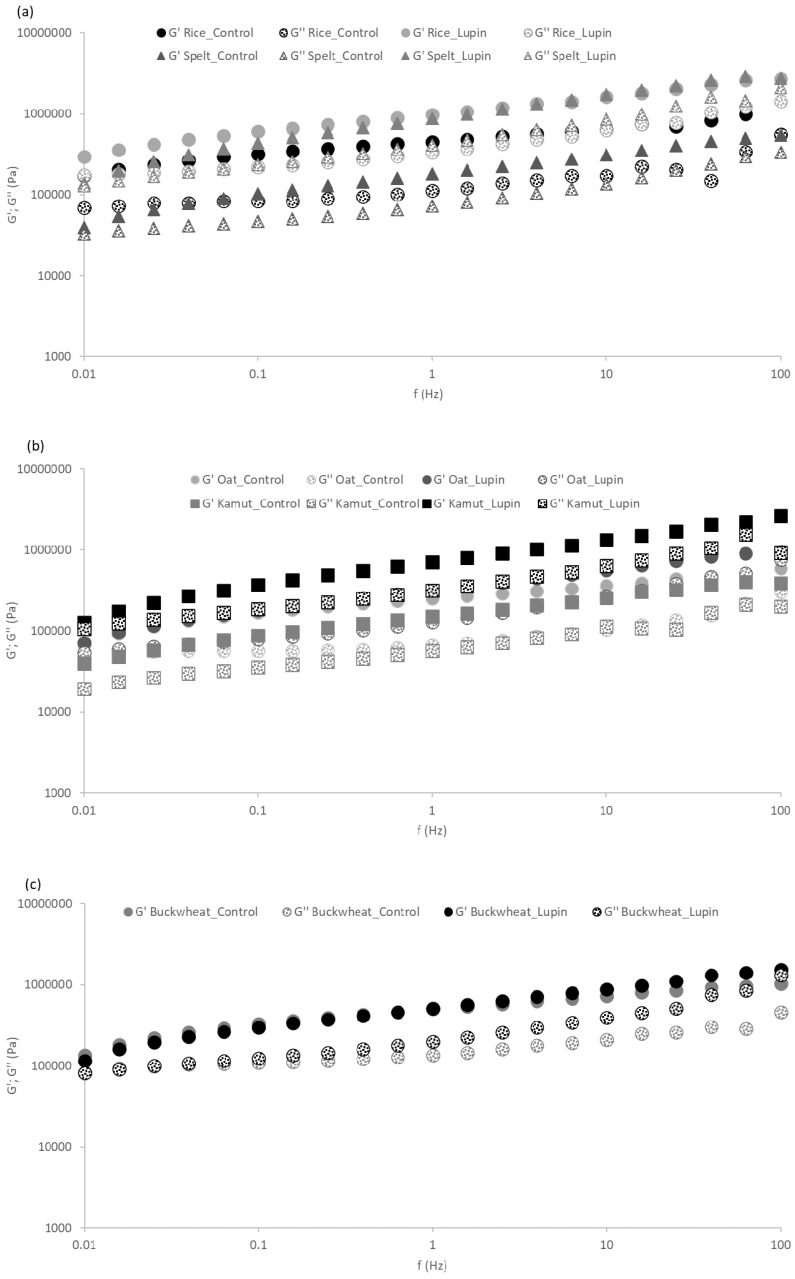
Mechanical spectra of the control and the lupin-enriched doughs prepared with five different flours: (**a**) rice and spelt; (**b**) oat and kamut; and (**c**) buckwheat. Solid symbols represent G’ (elastic modulus) and the dotted symbols represent G” (viscous modulus).

The impact of LE addition on the linear viscoelastic behavior of the cookie’s dough prepared with five different flours can be observed in Figure 2. These results were obtained from small amplitude dynamic rheological measurements (small amplitude oscillatory system - SAOS) and are related to the degree of dough structuring, reflecting the level of molecular interactions that are established, especially among the macromolecules present.

The evolution of G’ (storage modulus) and G” (loss modulus) over the frequency range tested reveal that both moduli slightly increased with increasing frequency. This weak gel-like rheological behavior is typical of cookie doughs [41] and other cereal dough products such as bread [42] and pasta [43].

The addition of LE causes the reinforcement of the dough structure for all the flours studied, except for the buckwheat flour. This is evidenced by the higher values of G’ and G” for the formulations enriched with LE, compared to the standard flours. These results are in agreement with the texture results-also in terms of firmness, the buckwheat flour formulation was the only one without significant differences (*p* > 0.05) due to the addition of LE.

To obtain a more detailed comparison among the linear viscoelastic behaviors of the different formulations, Table 2 shows the G’ values obtained at 1 Hz (G’ 1 Hz) from the three replicates of each test. It turns out that the G’ 1 Hz values were significant higher in rice, spelt and kamut flours, when the lupin incorporation dough was compared with the control without LE. The maximum value for G’ was 8.3 × 10^5^ Pa for the lupin-incorporated rice flour. However, the greatest increment measured in G’ 1 Hz due to the addition of LE was achieved for the kamut flour. In these cases, lupin incorporation increased the degree of dough structuring, which results from the formation of more complex three-dimensional structures among the macromolecules present in the systems, as previously discussed for the dough texture results.

### 3.2. Physical Properties of Cookies

Characteristic dimensions of the LE incorporation in five different flours are presented in Table 3. In general, the incorporation of LE had significant differences (*p* < 0.001) in all the flours tested, except for oat flour. For spelt, kamut and buckwheat flours, the addition of LE increased the area in relation to the control. However, the rice flour cookies were the only ones with a significant (*p* < 0.001) reduction in the cookie area. Therefore, the presence of gluten does not seem to affect the cookie area and no direct relationship can be established with the expansion of the structure. In relation to thickness, the two gluten-free flours (rice and buckwheat) showed a significant increase (*p* < 0.05) in the LE-containing cookies, unlike the gluten flours, where it showed a generalized decrease. Similar studies were performed with wheat cookies and Jayasena and Nasar-Abbas [26] reported no effect in the cookie diameter and an increase in the cookie thickness with the presence of 10% (*w*/*w*) lupin flour. Nevertheless, Bilgiçli and Levent [44] demonstrated no effect in the thickness in cookies containing lupin flour, whereas Tsen et al. [45] showed a reduction in the cookie diameter prepared with soy protein isolates.

In summary, even in cases where statistically significant results were obtained, all the structural alterations resulting from the addition of LE could be neglected, as far as the magnitude was concerned (maximum 20% variation for the area and thickness of buckwheat cookies). This conclusion can be important in terms of technological performance and consumer acceptance.

**Figure 3:**
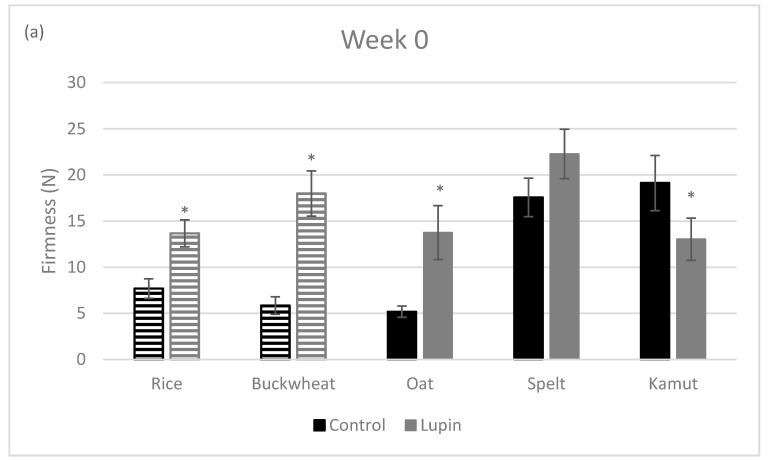

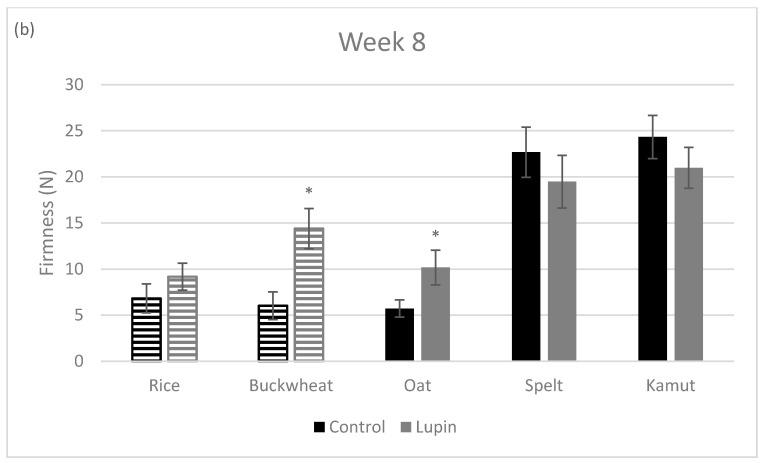
Firmness of the control and lupin-enriched cookies at week 0 (**a**) and week 8 (**b**). Solid bars represent the gluten-containing flours (oat, spelt and kamut) and the striped bars represent the gluten-free flours (rice and buckwheat). Values are the means of at least six experiments ± SD. * represents *p* < 0.05 when compared with the corresponding control cookie.

The texture properties of foods are an important requirement for their acceptance by consumers, especially in what concerns crispy products, such as cookies [46]. In this sense, the impact of LE addition to different types of cookies was evaluated in both the presence and absence of gluten. Firmness values (N) obtained in week 0 and eight weeks later are presented in Figure 3.

It is evident that the cookies prepared with the ancient grains and without LE (spelt and kamut) were firmer than the other control cookies and this observation remained valid after storage (eight weeks). The changes induced by the LE in the cookie structure differed over time, since at week 0 only the spelt flour had no significant difference (*p* > 0.05) when compared to the control; however, after eight weeks, the spelt, kamut and rice flours showed no significant differences (*p* > 0.05) when the cookie with LE and the control were compared. Additionally, the oat and buckwheat flours were statistically different over time (eight weeks), meaning that the incorporation of lupin clearly modified the texture of the cookies, making them firmer. Hence, it cannot be stated that the differences between the five different flours on one hand and LE addition on the other occurred due to the presence of gluten. Jayasena and Nasar-Abbas [26], Obeidat, Abdul-Hussain and Al Omari [47] and Bilgiçli and Levent [44] reported that cookie hardness increased with the addition of lupin flour in the cookie formulation. This can also be stated for other types of legume seeds, such as chickpeas [48], green lentils and navy beans [4]. The different behavior observed between the doughs and the respective cookies is corroborated with other studies [41]. Indeed, macromolecular structures present in each flour undergo dramatic changes during heat treatment. In spelt and kamut flours, gluten is the main element that accounts for the structure; in oat, the main structural role is played by β-glucans, and in gluten-free flours (rice and buckwheat), the structure is mainly accounted for by starch. When the LE (protein) is added, there is an overall structural rearrangement leading to distinct interactions among these macromolecules, as supported by our results. The interactions among macromolecules and the type of structures which arise are differentially affected by the heat treatment which takes place during cooking.

The impact of LE addition on the color parameters of cookies is summarized in Table 4. The ΔE* values were calculated to compare the color variation in relation to the cookies without LE. In the same table, the water activity (a_w_) and moisture content (H) of the ten formulations studied are also indicated.

The ΔE* values obtained were always higher than 5 for both time periods studied (week 0 and week 8), which means that the color difference between the lupin-enriched cookies and the control is visually distinguishable by the human eye. These differences result mainly from a general decrease in the lightness parameter (L*) in all lupin-containing cookie samples, resulting in a golden-brown color. These results agree with other studies, showing a decrease in cookie lightness with lupin flour at the same concentration level [44]. The results can be explained by the Maillard reaction, as proteins and sugars initiate a complex cascade of reactions during heating (higher than 100 °C), producing the darker color [49]. This darkening did not have a negative impact on the characteristics of the final product; on the contrary, the LE cookies presented very appealing colors, as those supported by other studies [26,28].

Cookies are a relatively dry product with a low moisture content and water activity values. These parameters are crucial to predict both the stability and safety of the product, with great impact in conservation, particularly for the maintenance of a crispy texture [50]. Moisture content values of cookies with and without LE are low (ranging from 1.04 to 5.61%), comparing favorably with other studies on similar cookies and indicating a positive impact in terms of conservation [30].

The a_w_ values for lupin-enriched cookies at week 0 are significantly higher (*p* < 0.05 or *p* < 0.001) than those of the control cookies. After 8 weeks of storage, all the LE cookies had similar (except for rice flour) a_w_ values, but significantly higher (*p* < 0.001) than the controls. Furthermore, all the samples were shown to have an a_w_ value of less than 0.5 (except lupin-enriched cookies with rice flour at week 0), which means that all cookie formulations (with and without LE) had a low percentage of free water for microbial proliferation, leading to a high stability product [50]. Such low a_w_ values are essential to prevent microbial growth on the cookies. Uysal et al. [51] found an increase in a_w_ values with the incorporation of apple and lemon fiber in cookies. Batista et al. [28] also found an increase in a_w_ values, resulting from the incorporation of microalgae biomass with a high protein content. However, Fradinho et al. [30] found an opposite effect when Psyllium fiber was added to the cookies similar to those prepared in the present work, resulting from the high-water holding capacity of Psyllium. The differential capacity to retain the water of the molecules present in the formulation had a direct impact on the water activity of the final product. For the LE cookies, the water holding capacity of the protein should be lower than that of the respective flour, justifying the increase in water activity.

Lupin is considered a potential functional food because of its protein content, dietary fiber and more recently discovered bioactivities [20,21] that need to be explored in food products in the near future.

## 4. Conclusions

Consumers are currently more cognizant about the environmental effects and nutritional benefits of foods. In this sense, lupin can be considered a suitable raw material for food production due to its nutritional and health-promoting properties.

Lupin protein extract (LE) addition to gluten and gluten-free flours showed a high impact in dough structure, increasing the degree of structuring. This impact on dough texture had technological implications, resulting in a greater difficulty in the molding process, which can be optimized in terms of industrial processing. The lupin-enriched dough based on buckwheat flour was unique because it did not show significant differences (*p* > 0.05) when compared to the control dough, being technologically more stable and easier to work with. Regarding the physical properties of the final products, the cookies based on buckwheat and oat flours were always firmer than the corresponding control cookies. Rice and buckwheat flours supplemented with LE produced cookies with a significant increase in thickness (*p* < 0.05), unlike in gluten flours (oat, spelt and kamut). These parameters are very important, since less thickness suggests more crispness, a highly desirable property appreciated by consumers. Supplementing flours with LE improves color and decreases lightness, making cookies more pleasant to consumers. After eight weeks, the a_w_ values of all LE-containing cookies were significantly higher (*p* < 0.001) than the controls, a characteristic which has a positive impact in conservation.

Overall, our results show that the cookies prepared with flours with or without gluten can be produced successfully by replacing 10% of the flour with LE. Therefore, the inclusion of 10% (*w*/*w*) sweet lupin protein extract in formulations improves the nutritional value and quality of cookies.

## Figures and Tables

**Table 1:** Nutritional composition of the five different flours used in the cookies’ formulations (g/100 g of flour). Values were provided by the suppliers Ceifeira and Próvida.

	Rice	Oat	Spelt	Kamut	Buckwheat
Energy (kcal/100 g)	350	370	295	385	366
Protein	7.6	14.0	13.0	15.0	13.3
Total lipid	0.7	7.6	1.8	15.0	3.4
Of which saturated	0.3	1.3	0.3	0.6	0.7
Total carbohydrate	78.5	56.0	55.0	60.0	61.5
Of which sugars	0.1	1.1	6.8	2.0	2.0
Of which fiber	2.4	10.0	4.0	11.0	10.0
Salt	<0.1	0.01	0.02	0.03	0.08

**Table 2:** Values of G’ when the frequency corresponds to 1 Hz. Values are the means of at least three experiments ± SD. * represents *p* < 0.05 when compared with the corresponding control cookie.

	G’ 1 Hz (Pa)	
	Control	Lupin
Rice	4.6 × 10^5^ ± 9.1 × 10^4^	8.3 × 10^5^ ± 1.3 × 10^5^ *
Buckwheat	5.0 × 10^5^ ± 2.9 × 10^4^	5.2 × 10^5^ ± 1.5 × 10^4^
Oat	2.5 × 10^5^ ± 8.3 × 10^4^	2.9 × 10^5^ ± 2.6 × 10^4^
Spelt	2.0 × 10^5^ ± 5.1 × 10^4^	6.2 × 10^5^ ± 9.6 × 10^4^ *
Kamut	1.6 × 10^5^ ± 1.4 × 10^4^	7.6 × 10^5^ ± 8.5 × 10^4^ *

**Table 3:** The dimensions of each cookie formulation with 10% (*w*/*w*) of lupin extract (LE). Values are the averages of ten cookies ± SD. * represents *p* < 0.05 and ** represents *p* < 0.001 when compared with the corresponding control cookie.

Cookie Formulation		Area (cm^2^)	Thickness (mm)
Rice	Control	15.63 ± 0.06	2.55 ± 0.14
	LE	15.08 ± 0.12 **	2.94 ± 0.08 *
Buckwheat	Control	13.26 ± 0.11	2.62 ± 0.11
	LE	16.05 ± 0.16 **	3.14 ± 0.10 *
Oat	Control	15.61 ± 0.10	3.25 ± 0.34
	LE	15.44 ± 0.21	2.79 ± 0.12
Spelt	Control	13.00 ± 0.03	3.57 ± 0.10
	LE	15.94 ± 0.11 **	3.35 ± 0.28
Kamut	Control	15.30 ± 0.07	2.94 ± 0.20
	Lupin	16.69 ± 0.06 **	2.68 ± 0.16

**Table 4:** Values of ΔE*, L*, a_w_ and the moisture content (H, % *w*/*w*) of the control and lupin-enriched cookies. Values are the means of at least three experiments ± SD, except ΔE* which is the difference between the control and lupin-enriched cookie colors. * represents *p* < 0.05 and ** represents *p* < 0.001 when compared with the corresponding control cookie.

		Rice	Buckwheat	Oat	Spelt		Kamut
ΔE*	Week 0		21.52	20.58	12.22	31.19	15.80
	Week 8		24.99	20.18	11.97	20.20	22.03
L*	Week 0	Control	80.40 ± 1.28	72.84 ± 0.81	71.27 ± 0.92	55.16 ± 3.04	73.43 ± 0.69
		Lupin	65.74 ± 2.73 **	56.78 ± 1.53 **	61.21 ± 1.47 **	64.68 ± 2.05 *	59.72 ± 1.11 **
	Week 8	Control	80.81 ± 1.06	69.37 ± 2.89	71.41 ± 0.97	78.43 ± 3.57	72.79 ± 1.70
		Lupin	62.10 ± 1.51 **	55.28 ± 3.02 *	63.11 ± 2.00 *	60.50 ± 2.21 *	53.41 ± 2.80 **
a_w_	Week 0	Control	0.36 ± 0.02	0.21 ± 0.01	0.04 ± 0.02	0.09 ± 0.01	0.50 ± 0.03
		Lupin	0.59 ± 0.01 **	0.35 ± 0.01 **	0.09 ± 0.00 *	0.40 ± 0.02 **	0.39 ± 0.01 *
	Week 8	Control	0.17 ± 0.00	0.12 ± 0.01	0.12 ± 0.00	0.22 ± 0.00	0.15 ± 0.01
		Lupin	0.23 ± 0.01 **	0.37 ± 0.00**	0.33 ± 0.00 **	0.36 ± 0.00 **	0.35 ± 0.00 **
H (%)	Week 0	Control	3.42 ± 0.14	5.61 ± 0.61	1.04 ± 0.11	2.75 ± 0.22	1.97 ± 0.08
		Lupin	2.88 ± 0.11 *	3.88 ± 0.08 *	2.29 ± 0.16 **	4.33 ± 0.08 **	2.85 ± 0.06 **
